# Supplementary material for: Computerized Cytological Features for Papillary Thyroid Cancer Diagnosis—Preliminary Report
Source: Cancers (Basel). 2019 Oct 25;11(11):1645. doi: 10.3390/cancers11111645 (PMC6896131; doi:10.3390/cancers11111645)
Supplement: Supplementary file 1 [file cancers-11-01645-s001.pdf]

Supplementary Material

Computerized Analysis of Cytologic Features

Digital cytological images in matrices of color pixels were collected for computerized analysis. The computerized analysis was performed using AmCAD-CA (AmCad BioMed Corp., Taipei, Taiwan). The detailed algorithm used by the software can be seen in the patent [1] filed by the software company. Briefly, the pixel values in the Red-Green-Blue (RGB) color space were first converted into the color space of hue (H), saturation (S), and value (V), with H_{ij}, S_{ij}, and V_{ij}, i=1,…, I and j=1,…, J, respectively, representing the H, S, and V values of the pixel at position (i, j) of the I×J matrix image. Based on color values, pixels were grouped using Otsu's method into 3 sets, i.e., nucleus, cytoplasm, and background sets [2, 3]. ***N, C***, and ***B*** denote the sets of nucleus, cytoplasm, and background, respectively, and n_N, n_C, and n_B denote the numbers of pixels grouped in ***N***, ***C***, and ***B***, respectively. The cytological features, including nuclear-cytoplasmic ratio (NCR), nuclear-cytoplasmic hue ratio (NCHR), nuclear-cytoplasmic saturation ratio (NCSR), and nuclear-cytoplasmic value ratio (NCVR), were then calculated using the following formulae

$$\text{NCR}=\frac{n_N}{n_C};\ \text{NCHR}=\frac{\sum_{(i,j)\in N} H_{ij}/n_N}{\sum_{(i,j)\in C} H_{ij}/n_C};\ \text{NCSR}=\frac{\sum_{(i,j)\in N} S_{ij}/n_N}{\sum_{(i,j)\in C} S_{ij}/n_C};\ \text{and NCVR}=\frac{\sum_{(i,j)\in N} V_{ij}/n_N}{\sum_{(i,j)\in C} V_{ij}/n_C}.$$

With the color pixels of cytoplasm and nuclei differentiated, the discrete nuclei were further segmented using the Canny edge detection method [4, 5]. The segmented margin of the nuclei could then be used for statistical values, such as the sample mean (M), the sample standard deviation (SD), and the coefficient of variation (CV=SD/M) of the morphological features including nuclear size, circularity, ellipticity, elongation, nuclear polarity, inclusion, and overlapping. With the total number of pixels within the margin of the kth discrete nucleus represented by n_k, where k=1,…, K, and K were the total numbers of segmented discrete nuclei, the mean nuclear size (MNS) and standard deviation of nuclear size (SDNS) were then calculated using:

$$\text{MNS}=\frac{\sum_{k=1}^{K} n_k}{K};\ \text{and SDNS}=\frac{\sum_{k=1}^{K}(n_k-MNS)^2}{K-1}.$$

With the perimeter of the kth discrete nucleus consisting of p_k pixels, the circularity of the kth nucleus was quantified as:

$$C_k=\frac{4\pi n_k}{p_k^2}.$$

The mean nuclear circularity (MNC) and standard deviation of nuclear circularity (SDNC) were calculated using:

$$\text{MNC}=\frac{\sum_{k=1}^{K} C_k}{K}\ \text{and SDNC}=\frac{\sum_{k=1}^{K}(C_k-MNC)^2}{K-1}.$$

With a and b as the long and short axis of the nucleus, the ellipticity of the kth nucleus was quantified as: $Ellip_k=\frac{4\pi n_k[3(a+b)-2\sqrt{ab}]}{abp_k}$.

The mean nuclear ellipticity (MNEllip) and standard deviation of nuclear ellipticity (SDNEllip) were calculated using:

MNEllip= $\frac{\sum_{k=1}^{K} Ellip_k}{K}$ and SDNEllip= $\frac{\sum_{k=1}^{K} (Ellip_k - MNEllip)^2}{K-1}$.

The elongation of the kth nucleus was quantified as:

$$Elon_k = \sqrt{1 - \left(\frac{b}{a}\right)^2}.$$

The mean of nuclear elongation (MNElon) and standard deviation of nuclear elongation (SDNElon) were calculated using:

MNElon= $\frac{\sum_{k=1}^{K} Elon_k}{K}$ and SDNElon= $\frac{\sum_{k=1}^{K} (Elon_k - MNElon)^2}{K-1}$.

For quantification, the area of the overlapped nuclei (n_{ON}) was first calculated by subtracting the total number of pixels in the nuclear area by the total number of pixels in the area of discrete nuclei:

$$n_{ON} = n_N - \sum_{k=1}^{K} n_k .$$

The overlapping index was then defined as the ratio of overlapped nuclei to the total nuclear area:

Overlapping Index (OI)= $\frac{n_{ON}}{n_N}$.

Similarly, to quantify cytoplasmic inclusion bodies, the number of pixels in the area of cytoplasm within nuclei (n_{CN}) was first calculated and the index was then defined as the ratio:

Inclusion Index (II)= $\frac{n_{CN}}{n_N}$.

For quantification of nuclear polarity, the angle of the long axis of the kth nucleus (θ_k) was first calculated. The index of nuclear polarity (NP) was then defined as the variability of nuclear long-axis angles and calculated using the sample standard deviation of θ_k.

Reference

1. Chen A, Hsiao YH, Chang TC, Jan IS, Shih SR & Wang HM. Cytological image processing device, and method for quantifying characteristics of cytological image. United States: Google Patents; 2018.
2. Fu KS & Mui JK. A survey on image segmentation. *Pattern Recognition*. 1981 **13** 3-16.
3. Jain AK. Data clustering: 50 years beyond K-means. *Pattern recognition letters*. 2010 **31** 651-666.
4. Bergmeir C, Garcia Silvente M & Benitez JM. Segmentation of cervical cell nuclei in high-resolution microscopic images: A new algorithm and a web-based software framework. *Comput Methods Programs Biomed*. 2012 **107** 497-512.
5. Canny J. A computational approach to edge detection. *IEEE Trans Pattern Anal Mach Intell*. 1986 **8** 679-698.
